# Facilitating the transition from physiology to hospital wards through an interdisciplinary case study of septic shock

**DOI:** 10.1186/1472-6920-14-78

**Published:** 2014-04-12

**Authors:** Albert S Li, Kenneth I Berger, David R Schwartz, William R Slater, David S Goldfarb

**Affiliations:** 1 NYU School of Medicine, New York, NY, USA; 2 Division of Pulmonary, Critical Care, and Sleep Medicine, NYU School of Medicine, New York, NY, USA; 3 André Cournand Pulmonary Physiology Laboratory, Bellevue Hospital, New York, NY, USA; 4 Leon H. Charney Division of Cardiology, NYU School of Medicine, New York, NY, USA; 5 Nephrology Section, VA New York Harbor Health Care System, New York, NY, USA; 6 Division of Nephrology, NYU School of Medicine, New York, NY, USA

**Keywords:** Integrated curriculum, Problem-based learning, Undergraduate medical education, Physiology

## Abstract

**Background:**

In order to develop clinical reasoning, medical students must be able to integrate knowledge across traditional subject boundaries and multiple disciplines. At least two dimensions of integration have been identified: horizontal integration, bringing together different disciplines in considering a topic; and vertical integration, bridging basic science and clinical practice. Much attention has been focused on curriculum overhauls, but our approach is to facilitate horizontal and vertical integration on a smaller scale through an interdisciplinary case study discussion and then to assess its utility.

**Methods:**

An interdisciplinary case study discussion about a critically ill patient was implemented at the end of an organ system-based, basic sciences module at New York University School of Medicine. Three clinical specialists—a cardiologist, a pulmonologist, and a nephrologist—jointly led a discussion about a complex patient in the intensive care unit with multiple medical problems secondary to septic shock. The discussion emphasized the physiologic underpinnings behind the patient’s presentation and the physiologic considerations across the various systems in determining proper treatment. The discussion also highlighted the interdependence between the cardiovascular, respiratory, and renal systems, which were initially presented in separate units. After the session students were given a brief, anonymous three-question free-response questionnaire in which they were asked to evaluate and freely comment on the exercise.

**Results:**

Students not only took away physiological principles but also gained an appreciation for various thematic lessons for bringing basic science to the bedside, especially horizontal and vertical integration. The response of the participants was overwhelmingly positive with many indicating that the exercise integrated the material across organ systems, and strengthened their appreciation of the role of physiology in understanding disease presentations and guiding appropriate therapy.

**Conclusions:**

Horizontal and vertical integration can be presented effectively through a single-session case study, with complex patient cases involving multiple organ systems providing students opportunities to integrate their knowledge across organ systems while emphasizing the importance of physiology in clinical reasoning. Furthermore, having several clinicians from different specialties discuss the case together can reinforce the matter of integration across multiple organ systems and disciplines in students’ minds.

## Background

Over the past decade, there have been several commissions calling for better integration of the basic and clinical sciences throughout all four years of undergraduate medical education in order to better prepare physicians for clinical medicine [1,2]. The dimensions of this integration are at least twofold: horizontal integration, which brings together different disciplines to consider a given topic (e.g., physiology and pharmacology in heart failure, or cardiology and nephrology in hypotension); and vertical integration, which applies basic science concepts to the assessment and management of a patient in a clinical scenario (e.g., using physiology to understand and treat a septic patient) [3]. Much has been published regarding various curricular approaches that increase the amount of integration, as well as the importance and effectiveness of horizontal and vertical integration [4-8].

Based on our experience in teaching physiology, we found that the organ system-based integrative approach was logical for our medical students and enabled them to immediately make connections between the various disciplines they were learning, for example, between physiology and pharmacology involving the heart. However, the organization of these disciplines by organ system did not lend itself to broader integration across the organ systems, a necessary skill in caring for more critically ill patients with complex problems involving multiple organ systems. To facilitate this further level of integration, a case study was developed to illustrate the interdependent physiology between the various organ systems and help students form these connections. Originally developed in business schools, case method teaching has been successfully used to discuss real-life, complex problems requiring, in medicine, a multidisciplinary approach, even integrating basic and clinical science in the preclinical years of medical school [9]. Unlike the problem-based learning approach, instructors take a more active role in guiding the discussion, which minimizes the prolonged pursuit of tangential topics [10]. Other medical educators have implemented patient cases that expose patients to clinical problem-solving to facilitate a smoother transition from the basic sciences to the clerkships [11].

This paper describes an exercise developed for the pre-clinical portion of our medical school curriculum to bridge the basic science and clinical curriculum by using these principles of horizontal and vertical integration. Through a demonstration of this multidimensional integration in action, we posited that students can begin to appreciate the need to think across the various subjects and apply this in considering a critically ill patient. The case-based discussion highlights how knowledge of fundamental physiologic principles must be used to appropriately diagnose and treat patients in the clinical setting. The case sought to build upon the existing cross-disciplinary connections of the basic sciences and help students begin to integrate their knowledge across various organ systems, which hopefully would continue throughout their medical training. The idea of integration would be emphasized further through the presence of three physician-facilitators—a cardiologist, a pulmonologist, and a nephrologist—and having students witness a discussion of the same patient from the point of view of those disciplines. Such an introduction to clinical decision-making in the basic science years would help prepare these medical students for their future clinical roles.

## Methods

The case study involved an interactive multidisciplinary discussion regarding a complex patient with septic shock that would be typically encountered in a critical care unit. The case was discussed with preclinical medical students at a private university medical school at the conclusion of their basic science coursework, 18 months into their medical school studies. The case and discussion questions were distributed to the students to review before attending an hour-long discussion session involving all the students in the class, jointly led by an interdisciplinary team of three physicians—a cardiologist, a pulmonologist, and a nephrologist. All three were the leaders of the physiology course sections for their respective organ systems. Students were not divided into smaller groups; rather, this exercise was held in a lecture hall that could accommodate the entire class. The three clinicians engaged in an informal conversation among themselves, describing their thought process about the patient’s disease state, while asking the students the previously distributed discussion questions. These questions served the purpose of providing students an opportunity to review what they had learned and synthesize it into an integrated understanding of this patient’s disease state. Other than the questions and the case itself, the session’s structure was left to the facilitators’ discretion. Facilitators were free to interject with important connections, as well as to challenge the other facilitators with questions of their own. Students were encouraged to pose questions as well and participate in this free-wheeling conversation.

This exercise was developed and first implemented in 2009 and used for each succeeding class of 160 students at the conclusion of their physiology coursework. Most recently in 2013, the exercise was conducted with a smaller group of 28 students, so that some subjective evaluative data could be gathered. The students were given a brief, anonymous three-question open-ended questionnaire in which they were asked to evaluate and freely comment on the exercise. The students’ free responses were evaluated by the faculty and summarized in Table 1. The responses to the first question, asking about take-away points, were categorized based on similar responses; specific answers about discrete principles or topics were classified as physiology concepts (e.g., Frank-Starling curve), whereas general ideas or problem-solving approaches were classified as themes (e.g., learning to integrate physiology across organ systems). Consistent with Code of Federal Regulations section 46.101, this work was considered exempt from requiring IRB approval as it constituted “research involving the use of educational tests (cognitive, diagnostic, aptitude, achievement), survey procedures, interview procedures or observation of public behavior” and included data collected without participant identification.

**Table 1:** Student responses evaluating the interdisciplinary case study of septic shock

	**Percentage of students**
***1. What are the main points you took away from this exercise?***	
**A. Themes**	
1. Understanding role of physiology in disease & clinical medicine	61%
2. Integrating physiology across organ systems (cardiovascular, pulmonary, renal)	57%
3. Learning to develop physiology-guided management/treatment plans	54%
4. Learning how to approach and think about patient presentation, including:	36%
a. Interpreting physical examination findings and lab values	
b. Formulating a differential diagnosis	
c. Working up a patient to confirm a diagnosis	
5. Highlighting topics in physiology to review before the wards (e.g., areas of weakness or importance)	25%
**B. Physiology Concepts**	
1. Frank-Starling relationship & volume status (underfilling versus overfilling)	46%
2. General overview of physiology	39%
3. Acid–base physiology	32%
4. Ventilation-perfusion mismatch	29%
5. Shock & hypotension	18%
6. Renal failure	7%
***2. Has this exercise changed your perspective concerning the importance of applying physiology in clinical medicine? If so, how?***	
Increased appreciation for role of physiology in clinical medicine	86%
***3. How useful do you believe this exercise is in preparing you for working on the medicine wards?***	
Useful/very useful	100%

**Figure 1:**
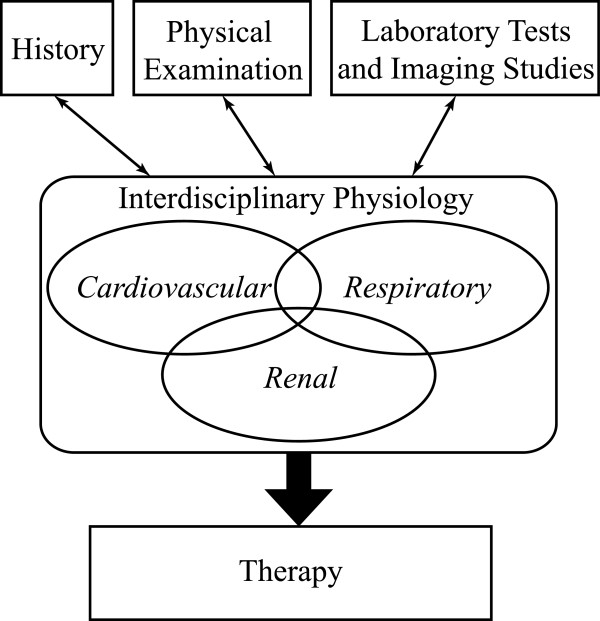
Role of physiology in clinical medicine.

The overall purpose of the case study was to demonstrate how a solid command of normal human physiology is crucial in bridging the physician’s assessment of the patient’s disease state through a thorough history and physical examination leading to the formulation of a treatment plan. This exercise requires not only an understanding of circulatory, respiratory, and renal physiology, but the integration of these systems in the context of a living person (Figure 1). The case and discussion questions are presented in the Appendix. In the discussion we provide several didactic points used by the physicians to integrate the students’ understanding of physiology across the various organ systems.

## Results

After the exercise, students were asked to evaluate the exercise along three lines: take-away points from the exercise, how the exercise changed their views of physiology in clinical medicine, and the usefulness of the exercise in preparation for clinical wards. The responses of the most recent group of 28 students who participated in this exercise are tabulated in Table 1.

The main points identified by the students were classified into two broad categories of themes and physiology concepts. All 28 students listed at least one point that was classified as a theme. These themes or general take-away points included understanding the role of physiology in clinical medicine, integrating physiology across organ systems, learning to use physiology to guide treatment, learning basic principles of clinical medicine (such as formulating a differential diagnosis, interpreting physical examination findings), and highlighting physiology topics to review in preparation for the wards. Interestingly, when asked for the take-away points from the exercise, 8 students (29%) did not identify any specific physiology concepts, focusing instead on listing some of the aforementioned themes or more general concepts. The fact that all of the participants mentioned these larger “themes” suggests that while the exercise did function as a review of physiology concepts for many students, overall they were able to appreciate the broader themes of applying physiology in clinical assessment and integrating their knowledge of organ systems, as opposed to merely thinking of them discretely.

Eighty-six percent of the students indicated that the exercise positively influenced their perspective concerning the importance of applying physiology in clinical medicine. All of the four students who did not believe the case study changed their views indicated that they already knew of the importance of physiology in clinical medicine; however, they acknowledged in their comments that the exercise reinforced its importance in their minds.

All of the participants indicated that they found this case study useful in preparing them for the wards. Although the sample size in this report is relatively small, this overwhelmingly positive reception by students has been consistent over the five years in which we have presented the case study but did not specifically solicit feedback about the case with a questionnaire. In several comments, students advocated for similar cases to be woven into their preclinical years, not because there was any lack of case studies but because this case integrated three systems together. One student remarked, “During the preclinical coursework, everything was taught and tested one system at a time…This exercise was a great integration. The wards will be a complete integration, which we have little experience doing.”

## Discussion

### Case study didactic points

#### Patient’s initial presentation and differential diagnosis

Developing a differential diagnosis, by definition, requires an interdisciplinary approach, in order to consider all the possible etiologies for a patient’s symptoms. The patient’s dyspnea, productive cough and pleuritic pain all suggested a pulmonary cause. The fever and chills, in addition to the elevated leukocyte count and immature white blood cells, pointed to a possible infection underlying the presentation.

This chief complaint of shortness of breath and fever however was extremely non-specific, which allowed for a discussion of the various etiologies that can lead to these two commonly encountered symptoms. Although the patient complained primarily of difficulty breathing, the cause was not necessarily in the lungs. Students were solicited for further questions to ask the patient in order to gather a more complete history and explore other etiologies. With some guidance from the physicians leading the conference, students were able to translate the history into a differential diagnosis by considering what diseases they were considering in asking these additional questions. One major possibility that was brought out was a cardiac origin for the pulmonary presentation, including processes such as congestive heart failure, endocarditis with septic emboli or myocardial depression secondary to infection. Further investigation was necessary to narrow down the wide range of possible diseases that could cause the patient’s presentation.

#### Blood pressure, cardiac output, and extracellular fluid volume status

The patient’s vital signs were consistent with systemic inflammatory response syndrome. In addition to the original complaint of fever and dyspnea, the patient was tachycardic and hypotensive. In considering the hypotension, the facilitators guided a discussion of the definition of blood pressure and various approaches to augmenting cardiac output. The three hemodynamic determinants of blood pressure—stroke volume, heart rate, and total peripheral resistance—were reviewed. Although the heart rate was high, the patient’s blood pressure remained low, pointing toward the stroke volume and total peripheral resistance as potential factors in causing the observed hypotension.

**Figure 2:**
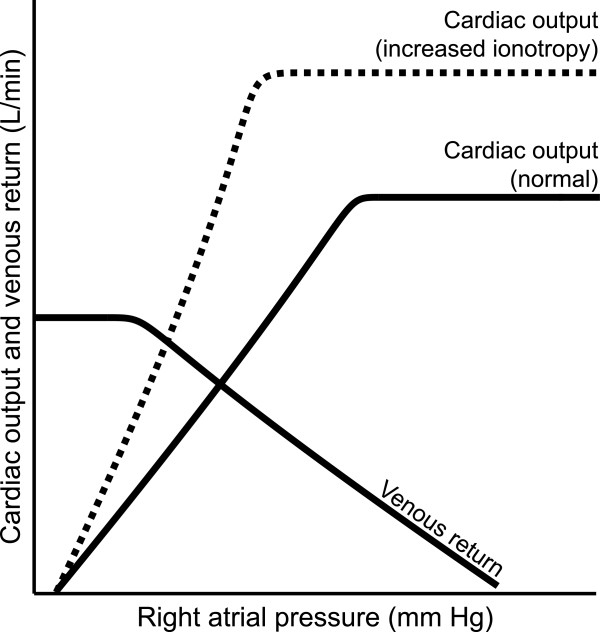
**Frank-Starling relationship.** Cardiac output and venous return are plotted as a function of right atrial pressure. Dotted line depicts shift in cardiac output due to increased inotropy. Adapted from Guyton [12].

When assessing stroke volume, an important judgment must be made on the patient’s position on the Frank-Starling curve (Figure 2). With an underfilled ventricle, for example, from intravascular volume loss, there will be less venous return to the heart, and a lower preload and cardiac output. Increasing the volume in the ventricle too much will overstretch the ventricle, requiring greater forces for the heart to contract completely (by Laplace’s law); without a change in cardiac contractility, the heart cannot sustain the optimal stroke volume, potentially leading to decreased cardiac output.

Determining whether a hypoperfused patient’s ventricle is underfilled or overfilled is important because the correct assessment dictates treatment. For a volume-depleted patient with an underfilled ventricle, the proper response would be to administer intravascular sodium-containing fluids. An increased intravascular volume would increase the left ventricular volume and increase cardiac output. However, administering fluid to an “overfilled” patient would be inappropriate; cardiac output would not increase as the overfilled ventricle is stretched even further with more fluid. In this scenario, intravascular volume must be reduced, which may seem counterintuitive in the presence of hypotension. Thus it is important to note that blood pressure is not itself an adequate marker of underfilling or overfilling.

The sign of rales, indicating alveolar fluid, is highly suggestive of pulmonary edema and an overfilled state. On the other hand, an inflammatory state such as pneumonia could also cause rales and pulmonary edema due to increased vascular permeability, making this finding less specific. Based on clinical experience, there are some septic patients early on in the “warm phase” who respond to volume. This phase is characterized by vasodilatation and an increased cardiac output. However, fluid administration can be overdone, leading to increased pulmonary capillary pressures and increased pulmonary edema. A fine balance exists between maintaining adequate tissue perfusion and preventing further pulmonary decompensation.

In addition to adjusting the patient’s extracellular fluid volume, another approach to optimizing cardiac output would be to increase inotropy. Drugs that increase contractility shift the entire Frank-Starling curve upward, increasing cardiac output for any given right atrial pressure (Figure 2). Examples of such positive inotropes include beta-adrenergic agonists, phosphodiesterase inhibitors, and digoxin. However, one major drawback to increasing inotropy is the greater oxygen demand it places on the heart.

Finally, diminishing afterload could increase cardiac output. The determinants of afterload are the internal radius of the ventricle and aortic impedance, which is related to blood pressure. Therefore, a peripheral vasodilator could be used to increase cardiac output. Clearly, using a peripheral vasodilator is only a theoretical option in a patient with borderline or low blood pressures, but it completes the conceptual framework of interventions to increase cardiac output. In summary, three ways to augment cardiac output are optimizing position on the Frank-Starling curve (Figure 2), increasing inotropy, and diminishing afterload, with each approach being better suited to different clinical scenarios.

#### Hypoxemia, acid–base balance

The patient’s hypoxia could have been solely due to inadequate perfusion due to low blood pressure, but poor blood oxygenation also contributed to inadequate peripheral oxygen delivery. Indeed, her blood oxygen saturation was lower than normal (92%), even after 100% oxygen was administered. The physical findings of tachypnea and rales, with the abnormal arterial blood gas, confirmed the presence of lung pathophysiology.

The arterial blood gas on 100% O_2_ showed acidemia (pH 6.98) and hypercapnia, with an elevated partial pressure of CO_2_ of 50 mm Hg. The arterial-alveolar O_2_ gradient (A-a PO_2_ gradient) was markedly above the limit for normal, suggesting that there was a problem intrinsic to the lung itself.

Radiologic studies suggested pulmonary edema or pneumonia. Fluid in the alveoli alters regional lung ventilation and impedes diffusion of gases between the alveolar air space and the pulmonary capillaries. The extremely high A-a PO_2_ gradient on 100% O_2_ indicated that there was anatomic shunting since O_2_ saturation was still suboptimal.

The arterial blood gas helped identify the etiology of this acid–base imbalance. Since the patient was hypercapnic with elevated CO_2_ levels in the blood, respiratory acidosis was present. However, the serum bicarbonate concentration was also markedly decreased at 10 mEq/L with an elevated anion gap. In this case, there was both primary respiratory and metabolic acidosis. The arterial blood gas showed an elevated lactate concentration, accounting for the anion gap. In the short term, lactic acidosis is adaptive because a decrease in serum pH would shift the hemoglobin-oxygen dissociation curve rightward and allow for increased delivery of O_2_ to tissues. However, without functioning lungs to oxygenate the blood, this is not a sustainable solution to tissue hypoxia.

## Conclusions

This hour-long case discussion introduced students to an integrated physiological approach to clinical decision-making, which would require further development during the clinical years and postgraduate medical training. Even in the course of a single session, students can begin to appreciate the need to think across the various subjects and apply this in considering a critically ill patient; this take-home message certainly left a deep impression on the first author who participated in this exercise as a student. This proof-of-concept study suggests that in the absence of a complete curricular overhaul, the principles of horizontal and vertical integration can be introduced in small steps with students still gaining some benefit from them.

In implementing this case or a similar case, educators and case facilitators need to assess the strengths and weaknesses of their students and curriculum and consider where points of integration are being missed, perhaps by virtue of how the curriculum is organized. Cases can then be adapted to complement the weaknesses of a particular curriculum, such as emphasizing connections between physiology and pharmacology at an institution in which these subjects are taught separately.

In addition to the usual requirements of dedication and preparation for case method teaching, the informal format described here obviously requires improvisation and a degree of personal and professional comfort and “chemistry” among the facilitators. The facilitators found that the degree to which they were relaxed was the degree to which students participated in the exercise, removing the stigma of yet another case discussion.

Based on student feedback, the exercise was well-received as an unconventional review exercise that was both educational and entertaining because of the informal, free-flowing, extemporaneous and even humorous format of the interactive dialogue between the clinicians. Positive assessments of efforts toward horizontal integration and vertical integration in the context of an entire curriculum have been reported by both teachers and students [5]. At the same time, the manner in which the clinicians took turns discussing the patient’s state helped reinforce the interconnections between the basic science disciplines and across the organ systems, bringing the focus to the level of the patient.

A limitation of this report is that students’ knowledge was not evaluated by objective methods in this study, but rather by self-reported subjective assessment. The authors considered that a short survey conducted immediately after the exercise would provide a better assessment of its utility, rather than the student’s performance on a clinical case, which would be dependent on many more factors. Ultimately the positive contribution of this short exercise to the real clinical thought processes of medical students reaching the hospital wards cannot easily be assessed. The feedback was quite positive but whether the exercise leads to a durable and more sophisticated approach to integrating physiology with clinical medicine is unknown.

One major advantage of having clinicians who both teach physiology and practice medicine lead the discussion was their ability to consider cardiovascular, pulmonary, and renal physiology in the context of how one affected the others in a clinical presentation, as opposed to considering them as discrete entities. In the course of studying such a difficult case, students were challenged to apply their existing understanding of physiology to analyze questions on a higher level than expected at their level of training. While some students found the complexity of the case frustrating, it served as an encouragement to review unclear physiological concepts before starting on the wards; they were also reminded that they would have many more opportunities to refine their clinical reasoning skills and employ physiological concepts to guide management. Ultimately, the students went away with an appreciation for the more sophisticated level of integration of one’s knowledge in considering complex patients with multiple medical problems.

## Appendix

### Case

#### Chief complaint

A delirious 44-year-old woman was brought to our emergency room complaining of shortness of breath and fever.

#### History

The patient had no past medical history until last week. She has been feeling “under the weather” for a couple of days. She presented a fever, a cough with some phlegm and chills. She also complained of some sharp chest pain when she took a breath. She denied smoking.

#### Physical examination

Physical examination revealed a patient with dyspnea in acute distress. Her temperature was 105 °F. Her blood pressure was 80/40 mm Hg (normally 120/80 mm Hg). Her heart rate was 140 beats per minute (normally 60–80 bpm) and regular. Her extremities felt cool to the touch and had mottled skin. She had decreased skin turgor.

After initial evaluation, she was put on 100% oxygen via a face mask. Even breathing 100% oxygen, her blood oxygen saturation was only 92% (normally >95% on room air and 100% on 100% inspired oxygen). Her respiratory rate was 35 breaths per minute (normally 15 breaths/min). Examination of the lungs revealed bilateral rales (crackly lung noises heard on inspiration that indicate fluid in the alveoli), which were more prominent at the bases. Her jugular veins were not distended and she had no hepatojugular reflux. The point of maximal impulse was in the fifth intercostal space along the left midclavicular line. She was tachycardic, with normal S1 and S2. There were no rubs, murmurs, or gallops. Peripheral pulses were weak on both sides. Her abdomen had some decreased bowel sounds but was otherwise normal.

#### Laboratory tests

After she was put on 100% oxygen, the laboratory assayed a sample of arterial blood from the patient and obtained the following values (normal values in parentheses): pH 6.98 (7.38-7.42), PCO_2_ 50 mmHg (37-43), PO_2_ 80 (>90 on room air), calculated HCO_3_ 10 (22-32), creatinine 2.5 mg/dl (0.5-1.5), blood urea nitrogen 40 mg/dl (5-25), Na^+^ 145 mEq/L (133-147), K^+^ 3.7 mEq/L (3.2-5.2), Cl^-^ 103 mEq/L (94-110), HCO_3_^-^ 12 mEq/L (22-32), lactate 5 mEq/L (<1.5).

Her white blood cell count was elevated (normal 4 – 12 × 10^3^ per microliter) and immature white cells were present. Her hemoglobin and hematocrit were normal, but her platelet count was low. Also, her urine output was low.

#### Chest X-ray

Chest X-ray showed pulmonary edema that was more pronounced at the bases and at the hila.

#### Computed Tomography (CT) scan

A lung CT scan showed extra density at the bases.

#### ECG

An ECG showed sinus tachycardia but was otherwise within normal limits.

#### Diagnosis and plan

The diagnosis was community-acquired pneumonia and septic shock. The patient was intubated and treated using lung protective ventilation. She was given vasopressors, antibiotics and normal saline.

### Discussion questions

#### Cardiovascular system

1. In the space below make a drawing of what this patient’s ECG might look like. Include at least two cycles and a time scale (remember to label each part).

2. Is this heart functioning on the ascending or descending limb of the Starling curve? How do you know, and what aspects of the exam, chest X-ray and other labs might help you decide? If we could insert a pressure manometer into each cardiac chamber, what data could you gain to help decide which limb her heart is on?

3. Suppose she is on the ascending limb because of fluid loss from sweating, blood drawing, and vomiting. How would we help her cardiac output?

4. Suppose she is on the descending limb. How should we treat her to increase her cardiac output?

5. Assume the contractility of her ventricles is only half normal; perhaps her blood pressure is low due to this. Alternatively, it could be low because of vasodilating endotoxins released from bacteria (she has a high fever). Could calculation of her systemic vascular resistance help in making this determination? What values do we need for this calculation?

6. Suppose you could increase her contractility by using our available drugs (beta agonists and phosphodiesterase inhibitors that increase cAMP or digitalis that inhibits the Na^+^/K^+^ pump). How do these help mechanistically? How about an investigational drug that acts on phospholamban or the ryanodine receptor?

#### Respiratory system

7. What is the most likely mechanism for hypercapnia in this patient? How would you determine if hypercapnia is due to reduced total ventilation or due to increased dead space? How could this information be applied to the clinical management of this patient?

8. What is the most likely mechanism for hypoxemia in this patient? How would you determine if hypoxemia is due to ventilation-perfusion mismatch or due to anatomic shunt? How could this information be applied to the clinical management of this patient?

9. What values do you expect for lung mechanics in this patient with acute respiratory distress syndrome (ARDS)? How could this information be applied to the clinical management of this patient?

#### Renal system and acid–base status

10. What is the acid–base disorder at the times when ABG (arterial blood gas) was done? What is the cause?

11. What is the differential diagnosis of this acid–base disorder in question 10?

12. Why does the acid–base disorder matter?

13. What affect does this scenario have on GFR? How is that change mediated?

14. What changes in sodium reabsorption occur concomitantly?

## Competing interests

The authors declare that they have no competing interests.

## Authors’ contributions

ASL drafted the manuscript and conceived the study of assessment of student response to the exercise. KIB helped in designing this case study, was one of the case facilitators, and helped to draft the manuscript. DRS helped in designing this case study. WRS helped in designing this case study and was one of the case facilitators. DSG helped in designing this case study, was one of the case facilitators, and helped to draft the manuscript. All authors read and approved the final manuscript.

## Authors’ information

ASL is a graduate of NYU School of Medicine who participated in this case as a medical student. KIB is an attending physician in pulmonary and critical care medicine, Associate Professor of Medicine, Physiology and Neuroscience at NYU School of Medicine and a member of the André Cournand Pulmonary Physiology Laboratory in Bellevue Hospital; KIB is also the physiology and pulmonary course director at NYU School of Medicine. DRS is an Assistant Professor of Medicine in the division of Pulmonary and Critical Care Medicine at NYU School of Medicine. WRS is an Associate Professor of Medicine at NYU School of Medicine, Co-Chief of the Cardiac Consultation Service at Bellevue Hospital, and former cardiology course director at NYU School of Medicine. DSG is a Professor of Medicine and Physiology at NYU School of Medicine, Clinical Chief of Nephrology at NYU Langone Medical Center, and Chief of the Nephrology Section at the VA New York Harbor Healthcare System; DSG is also the nephrology course director at NYU School of Medicine.

## Pre-publication history

The pre-publication history for this paper can be accessed here:

http://www.biomedcentral.com/1472-6920/14/78/prepub
